# Prediction and Analysis of Post-Translational Pyruvoyl Residue Modification Sites from Internal Serines in Proteins

**DOI:** 10.1371/journal.pone.0066678

**Published:** 2013-06-21

**Authors:** Yang Jiang, Bi-Qing Li, Yuchao Zhang, Yuan-Ming Feng, Yu-Fei Gao, Ning Zhang, Yu-Dong Cai

**Affiliations:** 1 Department of Surgery, China-Japan Union Hospital of Jilin University, Changchun, P. R. China; 2 Key Laboratory of Systems Biology, Shanghai Institutes for Biological Sciences, Chinese Academy of Sciences, Shanghai, P. R. China; 3 State Key Laboratory of Medical Genomics, Institute of Health Sciences, Shanghai Jiaotong University School of Medicine and Shanghai Institutes for Biological Sciences, Chinese, Academy of Sciences, Shanghai, P.R. China; 4 Department of Biomedical Engineering, Tianjin University, Tianjin Key Lab of Biomedical Engineering Measurement, Tianjin, P.R.China; 5 Institiute of Systems Biology, Shanghai University, Shanghai, P. R. China; University of Alberta, Canada

## Abstract

Most of pyruvoyl-dependent proteins observed in prokaryotes and eukaryotes are critical regulatory enzymes, which are primary targets of inhibitors for anti-cancer and anti-parasitic therapy. These proteins undergo an autocatalytic, intramolecular self-cleavage reaction in which a covalently bound pyruvoyl group is generated on a conserved serine residue. Traditional detections of the modified serine sites are performed by experimental approaches, which are often labor-intensive and time-consuming. In this study, we initiated in an attempt for the computational predictions of such serine sites with Feature Selection based on a Random Forest. Since only a small number of experimentally verified pyruvoyl-modified proteins are collected in the protein database at its current version, we only used a small dataset in this study. After removing proteins with sequence identities >60%, a non-redundant dataset was generated and was used, which contained only 46 proteins, with one pyruvoyl serine site for each protein. Several types of features were considered in our method including PSSM conservation scores, disorders, secondary structures, solvent accessibilities, amino acid factors and amino acid occurrence frequencies. As a result, a pretty good performance was achieved in our dataset. The best 100.00% *accuracy* and 1.0000 *MCC* value were obtained from the training dataset, and 93.75% *accuracy* and 0.8441 *MCC* value from the testing dataset. The optimal feature set contained 9 features. Analysis of the optimal feature set indicated the important roles of some specific features in determining the pyruvoyl-group-serine sites, which were consistent with several results of earlier experimental studies. These selected features may shed some light on the in-depth understanding of the mechanism of the post-translational self-maturation process, providing guidelines for experimental validation. Future work should be made as more pyruvoyl-modified proteins are found and the method should be evaluated on larger datasets. At last, the predicting software can be downloaded from http://www.nkbiox.com/sub/pyrupred/index.html.

## Introduction

The formation of an active pyruvoyl-dependent protein involves a self-maturation process via an autocatalytic post-translational modification (PTM). It has been observed spanning several decades in a number of often evolutionarily and structurally unrelated proteins in bacteria, Archaea and Eukarya [1]–[2]. Most of such proteins are critical regulatory enzymes in biosynthetic pathways, which are primary targets of inhibitors and are subjects of numerous biochemical and structural investigations for anti-cancer and anti-parasitic therapy [3]–[6].

Most well-studied enzymes that undergo such post-translational modifications catalyze either the decarboxylation or the reduction of amino acids and amino acid derivatives, with a pyruvoyl group as the enzyme cofactor [7]. L-aspartate-α-decarboxylase (ADC) converts L-aspartate to β-alanine, providing the major route of β-alanine production in the bacterial pantothenate-biosynthesis pathway [1], [8]–[9]. S-adenosylmethionine decarboxylase (AdoMetDC) is an essential enzyme for the biosynthesis of the polyamines spermidine and spermine, which are required for normal cell proliferation and differentiation. AdoMetDC catalyzes the conversion of S-adenosylmethionine (AdoMet) to S-adenosyl-5′-(3-methylthiopropylamine), as an early step in the polyamine biosynthetic pathway [6], [10]–[12]. Many bacteria or chlamydial strains have arginine decarboxylase (ArgDC) that converts L-arginine to agmatine [6], [13]–[15], used for a variety of metabolic or defensive purposes against host innate immune responses [16]. The histidine decarboxylation pathway consists of histidine decarboxylase (HisDC) that removes the α-carboxylate group of histidine, which causes histamine spoilage of traditionally fermented foods in food-borne bacteria, such as cheese and wine [14], [17]–[19]. Other examples of pyruvoyl group dependant enzymes are phosphatidylserine decarboxylase (PsDC) [20], glycine reductase, D-proline reductase [7], etc.

Such proteins show little similarities in sequence, structure, or oligomeric state; however the post-translational event would be similar in the enzyme maturation process, even in different species [2], [6]–[7]. They undergo an autocatalytic, intramolecular self-cleavage reaction in which a covalently bound pyruvoyl group is generated as its reactive cofactor. A conserved serine residue (Ser) is identified at the site of protein self-cleavage and pyruvoyl group formation in the decarboxylases. The post-translational modification follows an unusual pathway, termed as non-hydrolytic serinolysis [1]–[2], [13], [18], in which the hydroxyl group of the serine performs a nucleophilic attack at the carbonyl carbon atom of the preceding residue on the main chain, forming an ester intermediate. Cleavage of the ester intermediate results in two non-identical subunits. The subunit containing the N-terminal part of the uncleaved chain is called β-chain while the subunit containing the C-terminal part is called α-chain. The pyruvoyl group is formed at the N terminus of the α-chain, which is derived from the carboxyl end of the proenzyme while releasing NH_3_ by a two-step reaction with water [2]. In reductases, a cysteine residue rather than a serine is identified to be the precursor of the pyruvoyl group [1], [7]. However, in the present study, we only investigate proteins modified at serine sites, excluding those cysteine-modified reductases, since only 2 experimentally verified cysteine-modified proteins can be found in the Uniprot database in its current version.

Traditionally, the site of protein post-translational cleavage with pyruvoyl group formation is detected by experimental approaches [7], [20], which are often labor-intensive and time-consuming. With the increasing prominence of the modification, there is an urgent need for developing a computational method to rapidly and effectively identify the pyruvoyl residue site. Computational prediction of PTM sites has become a very important area in bioinformatics research community [21]. A number of different methods for predicting different types of PTM sites have been developed, such as protein phosphorylation site prediction [22]–[24], γ-carboxylation site prediction [25], methylation site prediction [26], lysine acytelation site prediction [27], glycosylation site prediction [28]–[29], S-nitrosylation site prediction [30] and many others. However, prediction methods for pyruvoyl residue sites are rarely developed. In view of this, the present study is initiated in an attempt to develop a new method to predict possible pyruvoyl serine sites with protein cleavage based on existing data. By using the Incremental Feature Selection approach based on several types of features such as PSSM conservation scores, disorders, secondary structures, solvent accessibilities, amino acid factors and amino acid occurrence frequencies, an optimal feature set is also provided.

## Methods

### Dataset

All proteins used in this study were taken from the UniProt database (release 2012_07, Jul 11, 2012) [31]. For rigorous evaluation of machine learning methods, it is important to use a non-redundant dataset. We removed those proteins with sequence identities >60% and those without experimentally verified pyruvoyl residues. Finally, a non-redundant dataset was generated containing 46 protein entries, the selected chains of which have less than 60% sequence identity.

Subsequently, by sliding a scaled window along each of the proteins, we extracted peptide segments with window length of 2*w*+1 centered on a serine residue, *w* residues upstream and *w* residues downstream of the serine site. The window length was set to be 15, 17, 19, 21, respectively. Peptide segments with length less than the window length were complemented by character “X”. Peptides with centered serine able to be formed pyruvoyl group were regarded as positive samples, while other peptides with centered non-pyruvoyl serine (‘non-pyruvoyl serine’ means ‘non-observed pyruvoyl serine’ in the this study) were as negative. Totally, 46 positive and 407 negative samples were extracted.

Since the dataset was extremely unbalanced with much higher number of negative samples, we randomly split the negative samples into four parts without overlapping, three of which had 102 negative samples and one had 101 negative samples. At each epoch we presented all the 46 positive samples, together with one of the four parts of negative samples (the ratio of positive/negative was about 1∶2). Thus 4 datasets were constructed which were numbered 1, 2, 3, 4, respectively. The number of samples in each dataset was given in **Table 1**. The 4 datasets were also provided in **File S1**. Each dataset was randomly separated into two parts for training and testing, respectively, with 4/5 of the data used for training and 1/5 for testing. 10-fold cross-validation test was adopted in the training process. The optimal features and the optimal window length providing the highest predictive performance were selected on the training set to build the final model. The testing set, which was not included in the training set, was then adopted to evaluate the model.

**Table 1:** The number of peptides in the 4 datasets used in this study.

	Positive peptides	Negative peptides	Total peptides
dataset 1	46	102	148
dataset 2	46	102	148
dataset 3	46	101	147
dataset 4	46	102	148
Total	46	407	453

### Feature Construction

The following features were utilized to encode every (2*w*+1)-residue peptide:

#### Features of PSSM conservation scores

Evolutionary conservation in the form of multiple alignments is considered important in biological sequence analysis [30]. A more conserved residue may play a more important role for the protein function. Herein, we computed position specific scoring matrix (PSSM) for each peptide and used as features (called PSSM conservation scores) to develop the prediction method. The PSSM profiles were obtained by using the Position Specific Iterative BLAST (PSI-BLAST) [32], a powerful sequence searching method, to search the UniRef100 database (Release: 15.10, 03-Nov-2009) through 3 iterations with 0.0001 as the E-value cutoff. For a specific residue in a peptide, a 20-dimensional vector was computed to denote the probabilities against mutations to 20 different amino acids. All such 20-dimensional vectors for all residues in a peptide composed a PSSM matrix.

#### Feature of disorder score

A protein region is defined as “unstructured” or “disordered” if it is devoid of stable secondary structure or if it has a large number of conformations. Such disordered regions could be quite important for the protein structure and function [33]–[34]. VSL2 [35], one of the best disorder predictor, was employed to calculate the disorder score for each residue in a given protein sequence. The disorder score ranges from 0 to 1, where the higher the score is, the more likely the residue lacks fixed structure.

#### Features of secondary structures and solvent accessibilities

Protein structure plays an essential role in deciphering its function. In addition, PTM of specific residues may be affected by their solvent accessibilities. In view of this, we also used features of secondary structures and solvent accessibilities to encode peptides. These features were predicted by SSpro4 [36].

In this study, the three types of secondary structures were denoted by a 3-bit binary string, i.e., ‘helix’ to ‘100′, ‘strand’ to ‘010′, and ‘other’ to ‘001’, respectively. And the two types of solvent accessibilities were encoded by another 2-bit binary string, i.e., ‘buried’ to ‘10’ and ‘exposed’ to ‘01’, respectively.

#### Features of amino acid factors

AAIndex [37] is a database containing various physicochemical and biochemical properties of 20 amino acids. Atchley et al. [38] performed multivariate statistical analysis on AAIndex and transformed AAIndex to five numeric attributes to reflect five properties: codon diversity, electrostatic charge, molecular volume, polarity and secondary structure. Here, we used these five numerical scores to represent the properties of each residue in a given protein sequence.

Note that since the residue at the center position in a peptide was always serine (Ser), it was not necessary to incorporate the amino acid factors.

#### Features of amino acid occurrence frequencies surrounding Ser

To investigate the position-specific amino acid compositions around the pyruvoyl serine site, we computed the occurrence frequencies of 20 native amino acids as well as the complemented element “X” in each position of the 2*w*+1 window length peptides in the training dataset. Results of the maximum window length 21 (containing results of other smaller window lengths) were shown in **File S2**. Since amino acid at the center site was always serine (Ser) in this study, it was not necessary to incorporate. And only the frequencies of the upstream and downstream sites were computed.

As mentioned above, features utilized in this study were summarized in **Table 2**. As seen in **Table 2**, for a peptide of length 2w+1, there were 20*(2w+1) PSSM conservation score features, 1*(2w+1) disorder score features, 3*(2w+1) secondary structure features, 2*(2w+1) solvent accessibility features, 5*2w amino acid factor features and 1*2w amino acid occurrence frequency features. In summary, a total of 64*w* +26 features can be extracted from a 2w+1 residue peptide. This kind of approach is quite similar to that used in [25] for predicting protein γ-carboxylation sites and that used in [30] for predicting protein S-nitrosylation sites.

**Table 2:** Features utilized to encode a (2*w*+1)-residue peptide.

Feature type	Features	Number	Sites
PSSM conservation scores	20-dimensional vector	20*(2*w*+1)	for all 2*w*+1 sites
Disorder score	Disorder score reflecting the disorder status of the residue	1*(2*w*+1)	
Secondary structures and solvent accessibilities	Secondary structures : helix, strand, other; Solvent accessibilities: buried, exposed	5*(2*w*+1)	
Amino acid factors	Polarity, secondary structure, molecular volume, codon diversity, electrostatic charge	5*2*w*	only for 2*w* surrounding sites (except the center)
Amino acid occurrence frequencies	Occurrence frequencies of 20 native amino acids as well as the complemented “X” in the peptide	1*2*w*	
Total		64*w*+26	

### Feature Selection

#### The mRMR method

The maximum relevance minimum redundancy (mRMR) method [39]–[41] was employed to rank the importance of the 64*w*+26 features. The mRMR method could rank the features according to their relevance to the target, and according to the redundancy among the features themselves. A ranked feature with a smaller index indicates that it has a better trade-off between the maximum relevance and the minimum redundancy.

To quantify both the relevance and the redundancy, the following mutual information (MI) is defined to estimate how one vector is related to another:
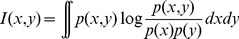
(1)where *x*, *y* are two vectors, *p(x,y)* is the joint probabilistic density, *p(x)* and *p(y)* are the marginal probabilistic densities.

Suppose *Ω* denotes the entire space containing all the aforementioned 64*w*+26 feature components, *Ωs* denotes the already-selected feature set containing *m* features, and *Ω_t_* denotes the to-be-selected feature set containing *n* features. The relevance *D* between the feature *f* in *Ω_t_* and the target *c* can be calculated by
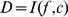
(2)


The redundancy *R* between the feature *f* in *Ω_t_* and all the features in *Ω_s_* can be calculated by
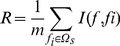
(3)


To get the feature *f_j_* in *Ω_t_* with the maximum relevance and the minimum redundancy, let us combine Eq. (2) with Eq. (3), as formulated by
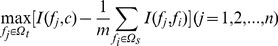
(4)


For a feature set with 64*w*+26 ( = *m*+*n*) components, the evaluation will continue for 64*w*+26 rounds. After these evaluations, a feature set *S* can be obtained by the mRMR method as formulated below:
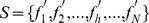
(5)where each feature in *S* has a subscript index indicating at which round the feature is selected. The better the feature is, the earlier it has been selected, and the smaller the index is.

#### Incremental feature selection (IFS)

Based on the ranked feature list evaluated by the mRMR approach, we used Incremental Feature Selection (IFS) [42]–[43] to determine the optimal feature set. In this study, during the IFS procedure, features in the ranked feature list were added one by one from higher to lower rank, i.e., 1, 2, 3, … A new feature set was constructed when another feature had been added. The i-th feature set is:
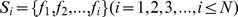
(6)


For each of the feature set *S_i_*, a predictor was constructed and examined. Thus, the optimal feature set could be obtained when the corresponding predictor yielded the best performance.

### Prediction Methods

#### The random forest method

The Random Forest (RF) algorithm [44], one of the famous machine learning methods, developed by Loe Breiman, has been successfully applied in various biological prediction problems [25], [45]–[46]. RF is an ensemble classifier consisting of several decision trees. Each tree is constructed according to the following procedure. (1) Suppose the number of cases in the training dataset is *N*, sample *N* cases at random. These samples compose the training set for growing the tree. (2) If there are *M* input variables, choose a number *m* which should be much less than *M*. At each node, *m* variables are selected randomly out of the *M* variables and the most optimized split on these *m* variables is employed to split the node. The value of *m* should keep constant during the forest growth. (3) Each tree should be grown to the largest extent possible without pruning.

To classify a new query sample, put its vector down each of the trees in the forest. Each tree yields a vote suggesting one class. The RF classifier will choose the class with the most votes over all trees. For detailed description about the RF algorithm, refer to [44], [47]. 10 trees were grown in our established RF model.

#### Performance measures

The efficiency of the method was assessed by 10-fold cross-validation test. This was a procedure in which the data set was randomly split into 10 equally-sized parts, each part being used in turn as testing set with the remaining 9 parts as training set. Thus the training and testing experiments were repeated for 10 times, and measurements were calculated as the average values of the 10 times experiments. In the training process, we used the 10-fold cross-validation test to evaluate the training performance.

Four measurements below were employed to evaluate the performance of the predictor. These measurements were derived from the four scalar quantities: *TP*, *FP*, *TN*, *FN*, which are the numbers of correctly predicted positive, incorrectly predicted positive, correctly predicted negative, incorrectly predicted negative samples, respectively.

*Accuracy (Ac)*, which is the fraction of correctly predicted sites among all the predictions, is calculated by:
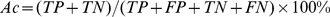



*Sensitivity (Sn)*, the rate of pyruvoyl serine sites that are correctly predicted as pyruvoyl serine sites, is given by:
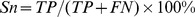



*Specificity (Sp)*, the rate of non-pyruvoyl serine sites correctly predicted as non-pyruvoyl serine sites, is given by:
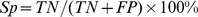



*Sn*, *Sp* and *Acc* stand for the success rates in positive, negative and overall datasets respectively. The fourth measurement, Matthews correlation coefficient (*MCC*), would be applied when the positive and negative datasets are unbalanced from each other:
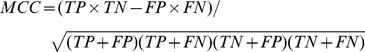



*MCC* is a number between -1 and 1. If there is no relationship between the predicted values and the actual values, the correlation coefficient is 0. A perfect fit gives a coefficient of 1.0. *MCC* was used throughout this study as the main evaluator for prediction performance. The optimal feature set could be obtained when the predictor achieved the best *MCC*.

#### Software implemented

In this study, the Random Forest classifier in Weka 3.6.4 [48] software was employed to perform the prediction. It was run with default parameters.

## Results

### The Optimal Window Length

**Figure 1:**
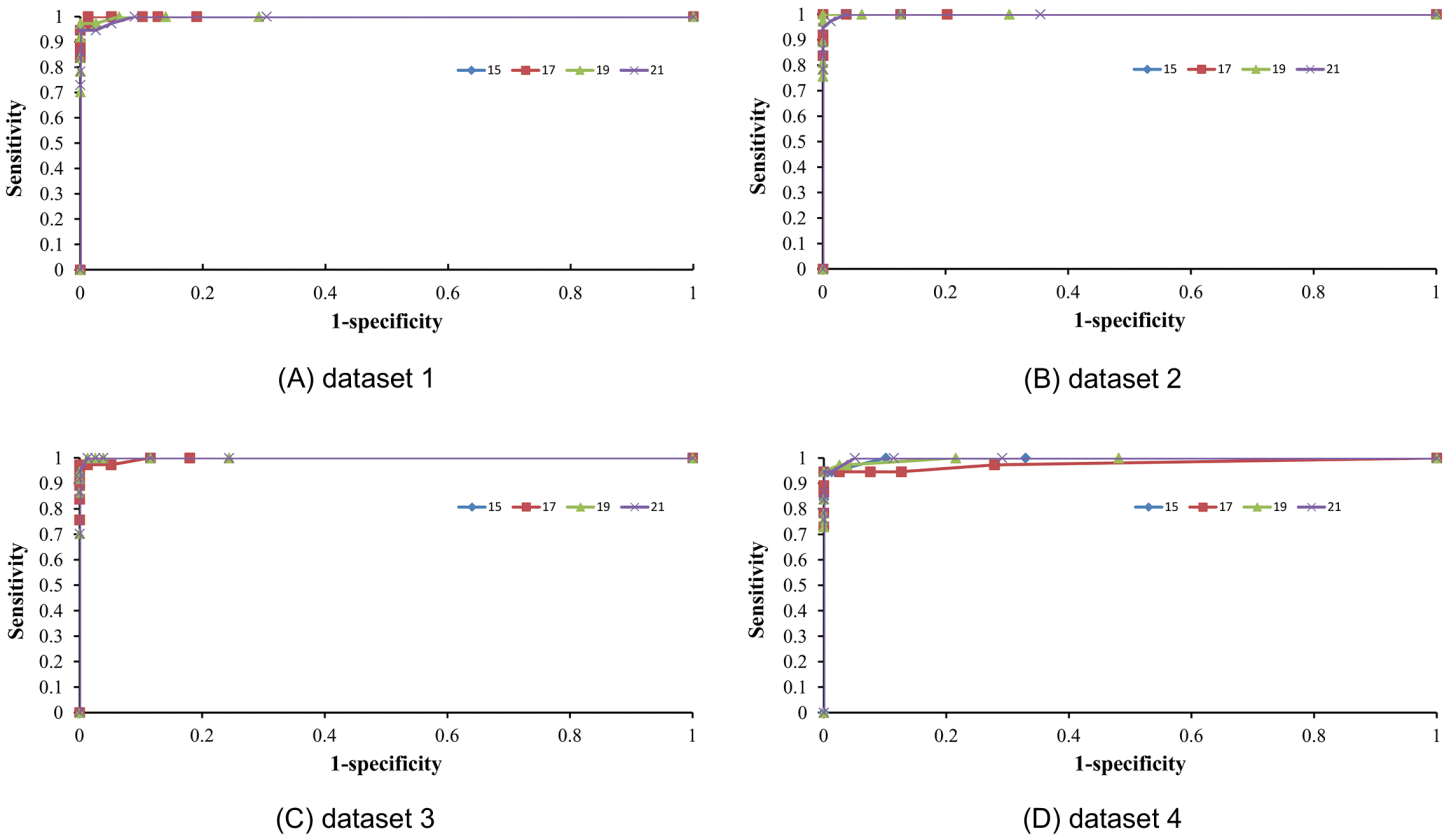
The ROC curves for 4 different window sizes in 4 datasets. Four plots showing ROC curves in the 4 datasets, respectively. Each curve was obtained when the highest *MCC* was achieved for each window size in each dataset. The corresponding number of features utilized for each curve was given in **Table 3**.

For each dataset, 4 different window sizes, i.e., 15, 17, 19, 21, were adopted. Therefore totally 16 iterations were preformed for the 4 datasets, each for one window size in one dataset. The predictive measurements and corresponding number of features selected were given in **Table 3** when the highest *MCC* was achieved in each of the 16 iterations. The corresponding ROC curves were depicted in **Fig. 1**. From **Table 3** and **Fig. 1**, it can be seen that similar performances can be obtained despite being trained on different datasets. The average *MCC* value reached the highest when the window length was set to 15 or 17 (**Table 3**). Therefore, in this study, 17 was regarded as the optimal window length (we selected the bigger size 17 rather than the smaller one 15).

**Table 3:** Highest training performance of each of the 4 different window lengths in the 4 datasets.

Window length	Dataset	Number of features	*Sn* (%)	*Sp* (%)	*Ac* (%)	*MCC*
15	1	8	100.00%	98.73%	99.14%	0.9805
	2	**9**	**100.00%**	**100.00%**	**100.00%**	**1.0000**
	3	13	97.30%	100.00%	99.13%	0.9801
	4	33	94.59%	100.00%	98.28%	0.9605
Average of 15			97.97%	99.68%	99.14%	0.9803
17	1	8	100.00%	98.73%	99.14%	0.9805
	2	**9**	**100.00%**	**100.00%**	**100.00%**	**1.0000**
	3	13	97.30%	100.00%	99.13%	0.9801
	4	15	94.59%	100.00%	98.28%	0.9605
Average of 17			97.97%	99.68%	99.14%	0.9803
19	1	24	97.30%	100.00%	99.14%	0.9802
	2	37	97.30%	100.00%	99.14%	0.9802
	3	12	100.00%	98.72%	99.13%	0.9804
	4	45	94.59%	100.00%	98.28%	0.9605
Average of 19			97.30%	99.68%	98.92%	0.9753
21	1	34	94.59%	100.00%	98.28%	0.9605
	2	34	94.59%	100.00%	98.28%	0.9605
	3	12	100.00%	98.72%	99.13%	0.9804
	4	32	94.59%	100.00%	98.28%	0.9605
Average of 21			95.94%	99.68%	98.49%	0.9655

### The mRMR Results

In each of the 16 iterations, two tables were obtained after running the mRMR software. One was called MaxRel feature table that ranked the 64*w*+26 features according to their relevance to the class of samples; the other was called mRMR feature table that ranked with the maximum relevance and the minimum redundancy to the class of samples. In the mRMR feature table, a feature with a smaller index implied that it was a more important one for pyruvoyl-serine site prediction. Such a list of ranked features was to be used in the following IFS procedure for the optimal feature set selection. The mRMR results of window length 17 for the 4 datasets can be found in **File S3**, data not shown of other window lengths since the optimal size was selected to be 17.

### The Prediction Results

**Figure 2:**
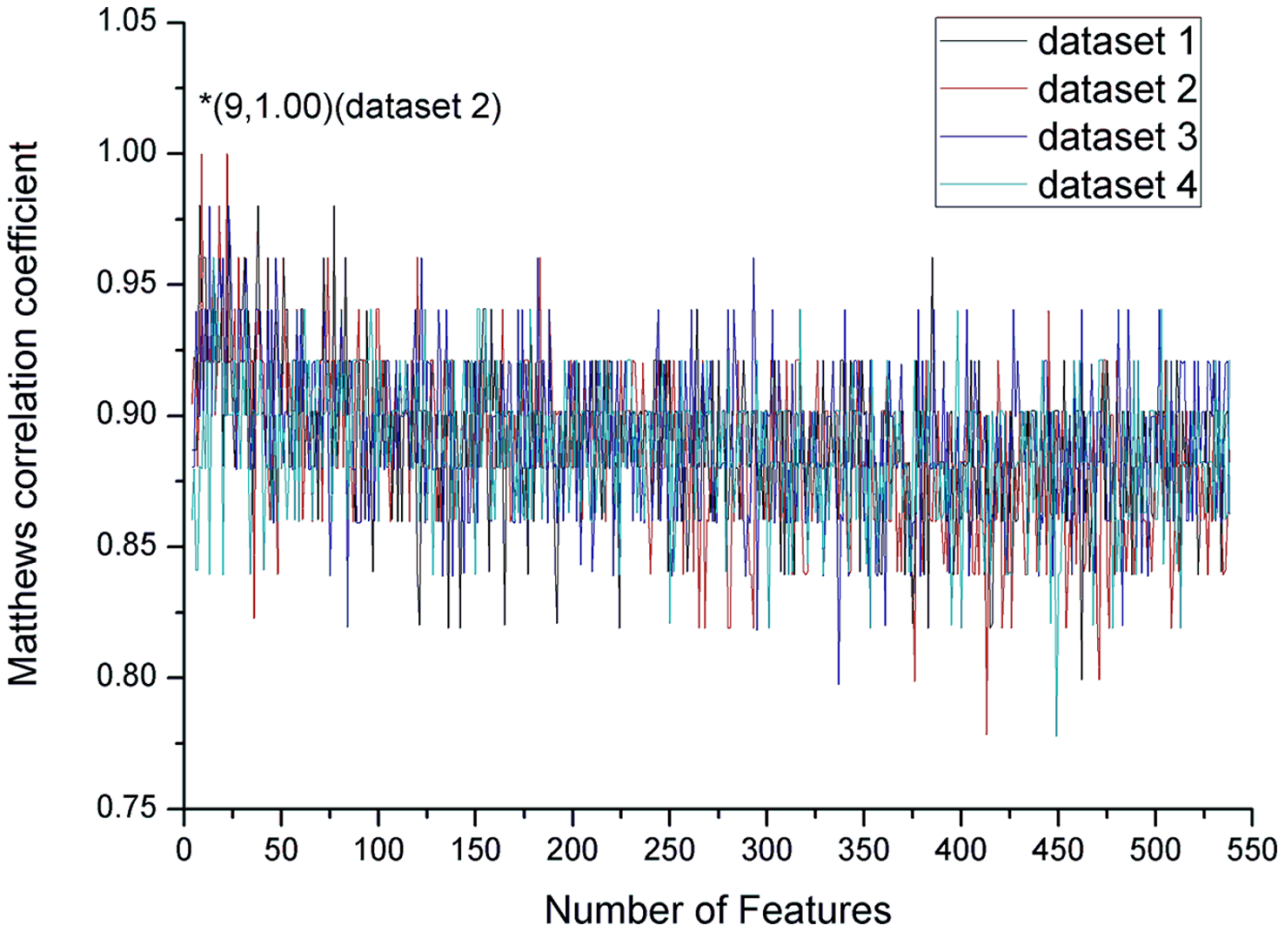
*MCC* values of predictors against different number of features selected when the window length was set to 17. Plot to show the *MCC* values against different number of features selected during the Incremental Feature Selection process. When the first 9 features were selected from the mRMR feature list, a peak of *MCC* (1.0000) was obtained in dataset 2. These 9 features were regarded as composing the optimal feature set for the pyruvoyl serine prediction.

In each iteration, by adding the ranked features one by one from the 64*w*+26 features in the mRMR table, we obtained 64*w*+26 different feature sets and accordingly built 64*w*+26 individual predictors. We then tested the prediction performance for each of the 64*w*+26 predictors and obtained the IFS results. Results of 17-residue peptides in the 4 datasets were given in **File S4**, data not shown of other window sizes. The IFS curves were shown in **Fig. 2**, which were plotted based on the data of **File S4**. It can be seen that the prediction results of window size 17 in the 4 datasets were slightly different from each other, suggesting the prediction model built on window size 17 was stable despite being trained on different datasets.

As we can see from **Fig. 2**, the *MCC* reached its maximum (1.0000) when the first 9 or first 22 features were selected. Such 9 features (we chose the smaller one 9 rather than 22) were regarded as composing the optimal feature set (the 9 features can be found in the mRMR table in **File S3**), and were used to construct the final model by applying a window length 17 for our pyruvoyl-serine prediction in this study. Performances of the model evaluated on the testing sets of the four datasets were given in **Table 4**. It can be seen from **Table 4** that the final model obtained a pretty good performance of accuracy 93.75% and 0.8441 *MCC* value. The excellent *Specificity* denoted that the method had a strong ability to distinguish the non-pyruvoyl serine sites from real non-ones. However, the *Sensitivity* was a little lower, which indicated that the ability of distinguishing the pyruvoyl serine sites from real ones was a little weaker.

**Table 4:** Performances of the final model evaluated on the four testing datasets (Window length = 17, 9 features used).

Testing Set of Dataset	*Sn* (%)	*Sp* (%)	*Ac* (%)	*MCC*
Dataset 1	88.89%	95.65%	93.75%	0.8454
Dataset 2	88.89%	95.65%	93.75%	0.8454
Dataset 3	77.78%	95.65%	90.63%	0.7624
Dataset 4	88.89%	100.00%	96.88%	0.9230
Average	86.11%	96.74%	93.75%	0.8441

### Distribution of the Optimal Features

**Figure 3:**
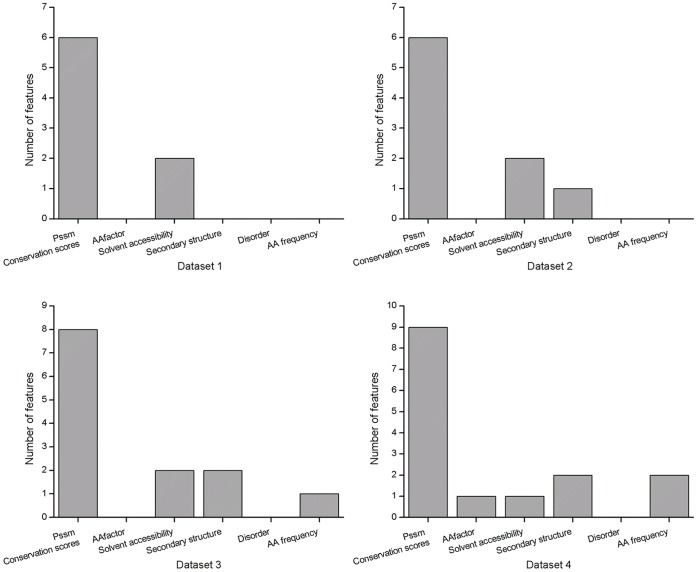
Histograms showing type distributions of the optimal features selected from the 4 datasets, respectively. The optimal features were selected when the MCC value reached its maximum trained on 17 length peptides in the training set of the 4 datasets, respectively.

We selected the first 9 features in the mRMR feature list obtained from dataset 2 as the optimal feature set. However, different optimal feature sets can be selected from other datasets, i.e. 8 features from dataset 1, or 13 features from dataset 3, or 15 features from dataset 4, as shown in **Table 3** (detail of the features can be found in **File S3**). We analyzed the feature type distributions of the different optimal features from the 4 datasets, with results depicted in **Fig. 3**. It is obvious that although the optimal features from 4 datasets were not all the same, the type distributions were slightly different. It can be seen from **Fig. 3** that the PSSM conservation scores affected the most. The secondary affecting feature types were solvent accessibilities and secondary structures. Therefore, we selected the 9 features from dataset 2 as the optimal features to construct our prediction model.

**Figure 4:**
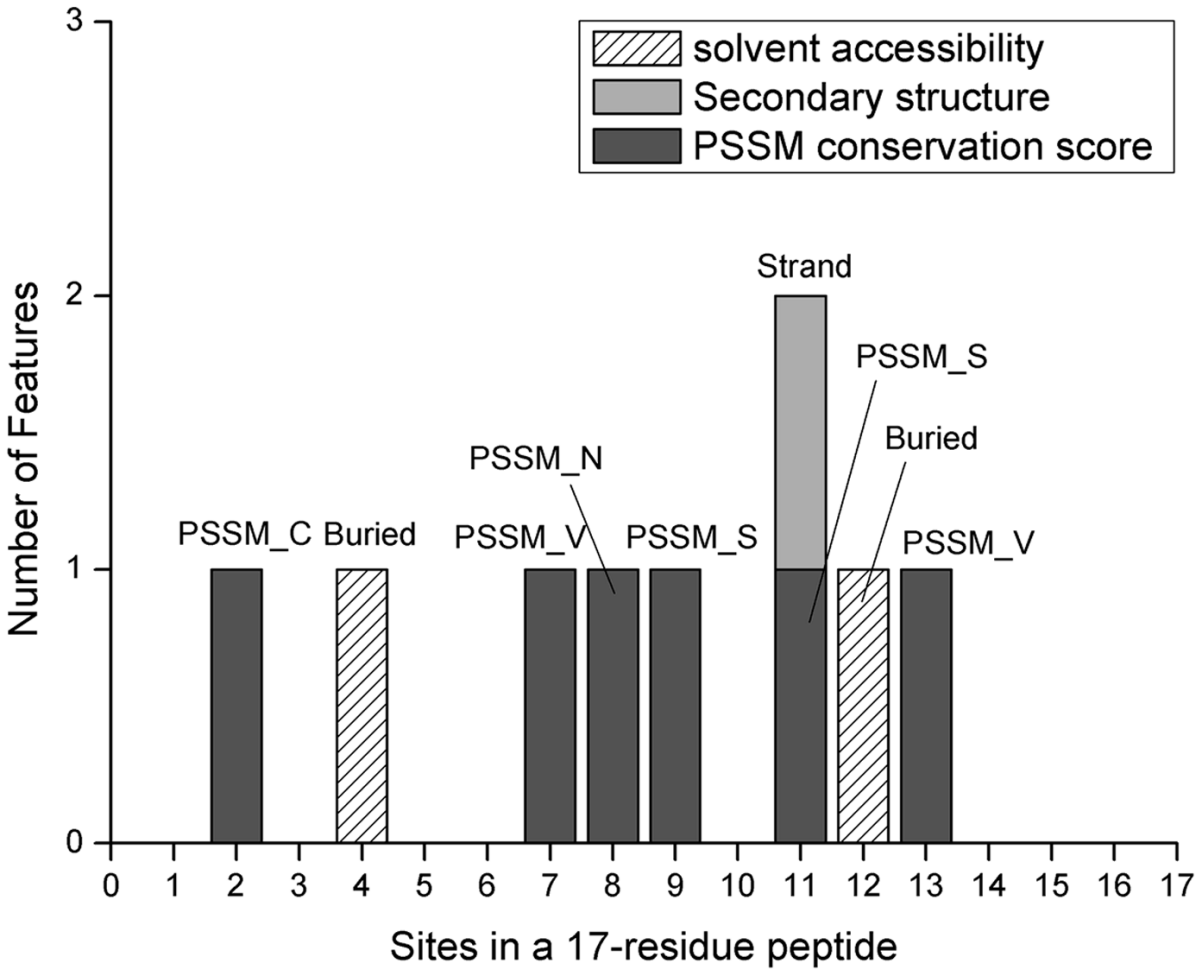
Histograms showing site distributions of the optimal features selected from dataset 2. Gray bars, light gray bars and hatched bars represented features of conservation scores, secondary structures and solvent accessibilities, respectively. Text above each bar indicated the corresponding feature.

Site distributions of the 9 features were shown in **Fig. 4**. It was revealed from **Fig. 4** that features selected from sites 2, 4, 7, 8, 9, 11, 12, 13 affected, suggesting residues at those sites played more important role in the determination of the pyruvoyl modification. There were 9 sites 1, 3, 5, 6, 10, 14, 15, 16, 17 having no features selected in our prediction method. Almost all feature-selected sites except 4 and 12 had one PSSM feature, indicating the important role of the amino acid conservations to the pyruvoyl-serine modification. Although slightly less relevant, it can be seen from **Fig. 4** ****that features of secondary structure and solvent accessibility also affected, suggesting the protein structures also played some roles as well as amino acid conservations in the protein pyruvoyl serine self-processing. These affecting factors will be further discussed in the following section.

### Amino Acid Occurrence Frequencies

**Figure 5:**
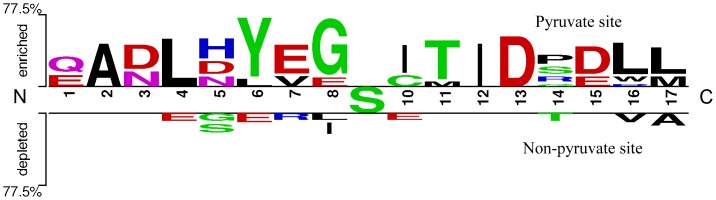
Amino acid occurrence frequencies surrounding the active-serine generated by WebLogo. The logo illustration was generated from the 17-residue peptides in the training set of dataset 2, showing the occurrence frequencies of amino acids surrounding the active serine. N and C represented the N- and C-terminuses of the 17-residue peptides, respectively.

We calculated the occurrence frequencies of 20 native amino acids (as well as “X”) for the 17 length positive peptides and negative peptides respectively in the training set. Results were depicted with a WebLogo (http://weblogo.berkeley.edu/) in **Fig. 5**. It is obvious from **Fig. 5** that the occurrence frequencies of the positive and negative peptides were different, indicating pyruvoyl-peptides had specific features that can be distinguished from non-pyruvoyl-peptides.

## Discussion

### Optimal Feature Analysis

From **Fig. 4**, it is demonstrated that the optimal feature set contained 6 PSSM conservation score features, 2 solvent accessibility features and 1 secondary structure feature. The 2 solvent accessibility features were all Buried features, indicating the protein structure affected. The selected 1 secondary structure feature was Strand, suggesting β-strand was related to pyruvoyl serine formation. None of amino acid factor, disorder and amino acid occurrence frequency features was selected, indicating those types of features not only have low relevance to pyruvoyl serine formation, but also contribute little to the prediction.

In our prediction method, no features of amino acid occurrence frequencies were selected. This suggests that the composition of the 20 amino acids surrounding the center serine could not play so much important roles in the determination of pyruvoyl serine modification. The post-translational pyruvoyl residue modification is considered as an autocatalytic procedure [6], [9]–[10], in which a protein self-cleaves at conserved serine residue to form an amino-terminal β-subunit and a carboxyl-terminal α-subunit with a reactive pyruvoyl cofactor. It could be conceived that a recognition site may be not so important as that in other proteinase-required post-translational modifications. For example, the post-translational γ-carboxylation of Glu in a protein requires a γ-carboxylation recognition site, called γ-CRS, to be bound to the carboxylase to perform the reaction [25]. However, different from γ-carboxylation, no recognition site may exist in a pyruvoyl residue modification. This was consistent with the result that no disorder feature was selected and the previous findings that intrinsic disorder was strongly correlated with proteinase-required post-translational modifications [49]. Since disorder regions affect binding sites, they are not requirements in pyruvoyl residue formation as an autocatalytic process.

### Evolution Features Play a Key Role in Pyruvoyl Serine Prediction

In the optimal 9 features, 6 belong to the PSSM conservation scores. In addition, although the 2 solvent accessibility features and 1 secondary structure feature were selected in the optimal feature set, indexes of them were above 70 in the MaxRel feature list (see **File S3**). And in such a list, all the top 27 features were PSSM conservation scores. It is indicated that amino acid conservations play the most important role in the pyruvoyl group formation and protein self-cleavage modification. It was also conceivable that in principle mutations to different types of amino acids had strong impact on the post-translational modifications of pyruvoyl-dependent enzymes. These observations from this study also supported the hypothesis of convergent evolution in the creation of their similar functions such as catalyzing amino acid decarboxylation reactions [2], and strongly drew that evolution information acted as an irreplaceable role for the prediction of pyruvoyl residue sites.

However, effects of mutations to different amino acids were different as seen in **Fig. 4**. Mutations to S, V affected the most (2 features), while mutations to C, N affected weaker (1 feature). Several important sites can also be found in **Fig. 4**, which suggested that mutations occurred at sites 2, 7, 8, 9, 11, 13 affected the most. It can also be seen that glycine (Gly, G) residue accounted for the most at site 8 in **Fig. 5**, where different situations can be found for non-pyruvoyl peptides. This was consistent with the general agreement of earlier studies that Gly is the most common residue preceding the nucleophile in self-processing systems, presumably due to its conformational adaptability [1]. However, in this study, other potential important mutation sites were also suggested, as complement to the previous findings. In spite of this, the discovery may be helpful and need to be experimentally studied.

Such highly conserved pyruvoyl peptides were thought to be one of the reasons on why the perfect prediction performance was obtained in this study (100.00% *accuracy*, 1.0000 *MCC* for training, and 93.75% *accuracy*, 0.8441 *MCC* for testing). From **Fig. 5**, it is observed that residues around pyruvoyl serine sites are highly conserved, significantly different from that in non-pyruvoyl peptides, which was consistent with previous studies. It is indicated that there is a strong signal difference in the two types of peptides that machine learning algorithms can efficiently extract. Although the dataset used in this study was small, results obtained from a non-redundant dataset can reflect the rules of such type of pos-translational modification. Therefore, it is expected to provide good generalization performances even on larger datasets. However, future work should be made as more pyruvoyl-modified proteins are experimentally verified and the method should be evaluated on those newly observed proteins.

### The Pyruvoyl Residue Modifications are Correlated with β-strands

Besides the PSSM conservation score features, the solvent accessibility and the secondary structure features were also compiled. In **Fig. 4**, it was obvious that only strand had impact on the modification, neither helix nor coil structures affected.

**Figure 6:**
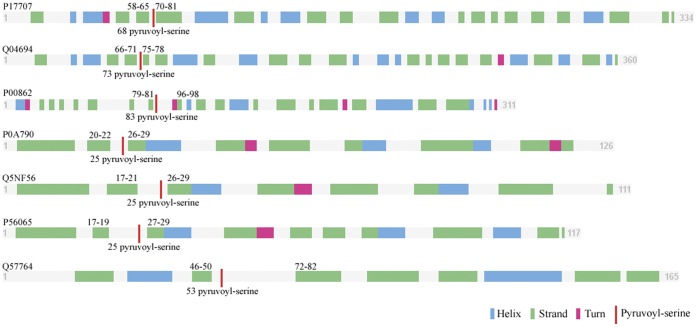
Secondary structures and the pyruvoyl-serine sites of the 7 structured proteins in our dataset. Secondary structures are represented by different coloured rectangles of length proportional to the length of the structure region. The pyruvoyl-serine site is depicted as a red line in the sequence. Initial and ending residue numbers of β-strands surrounding the active-serine sites are printed above the sequence. Residue numbers of the active-serine sites are printed below.

In the present study, we investigated all the pyruvoyl-serine sites and the surrounding secondary structures of proteins in our dataset. Totally 7 proteins in our dataset had experimentally-determined structures in the Protein Data Bank (PDB). The secondary structures of the 7 proteins and the pyruvoyl-serine sites were depicted in **Fig. 6**. Dramatically, it can be seen from **Fig. 6** that all the 7 proteins undergo a post-translational modification at a site between two β-strands, and all the modified serine sites are in a no regular secondary structure region.

The preference of the location of a self-cleavage site in a loop between two β-strands or in close proximity to a β-strand has been suggested by several previous studies [1]–[2], , despite different pyruvoyl-dependent proteins show little similarity in sequence, structure, or oligomeric state. Schmitzberger et al. [1] suggested the determination role of the conformational freedom in the loop preceding the cleavage site in the PTM reaction of ADCs. They also found that longer loop would render the loop incapable of stabilizing intermediates from studies of insertion mutants, suggesting a certain degree of conformational flexibility as an important factor. Bale et al. [6] suggested that residues close to the loop may play a role in stabilizing the oxyoxazolidine intermediate in the autoprocessing reaction. β-strand impacts would also be a general rule for protein cleavage, both for protease-required reactions and for self-cleavage reactions [50], [51]–[53]. It can be realized that β-strand structures play an important role on pyruvoyl residue modifications, and a serine needs a no-regular secondary structure to perform the nucleophilic attack.

Many amino acids of top-most maximum frequencies at specific sites can be found in **Fig. 5**, except the center serine and the preceding G, which were discussed above. It is interesting to see that almost all top-most frequency amino acids are β-strand favorite residues [54]–[56], such as L, I, Y, T, E, A. And different situations can be found for non-pyruvoyl peptides in **Fig. 5**. These observations could be one of the reasons to explain the discrepancy that why none amino acid frequency features were selected while obvious high-frequency residues can be found in **Fig. 5**. These high-frequency residues were not composed as a consensus sequence of pyruvoyl formation, but as important residues to form β-strand structures surrounding the cleavage sites. Another reason could be feature redundancy may exist between amino acid frequency features and the selected features. They were removed from the optimal feature set by applying the Minimum Redundancy criterion.

### Additional Factors

The performance of the predictor developed in the present study was quite good, however the *Sensitivity* was a little lower, which indicated that the weaker ability of distinguishing the positive pyruvoyl-serine sites from real ones. Reasons would be the additional factors beyond those in the 17-residue peptides [7]. Webb et al. [9] indicated that the β-amide of Asn72 was hydrogen bonded to the β-hydroxyl group of Ser25 in the unprocessed structure of ADC, holding the latter residue in an unreactive conformation, whereas Asn72 was not required for activation of the protein. Trip et al. [18] showed that the protein HdcB encoded by hdcAPB operon of *S. thermophilus* was involved in the post-translational pyruvoyl modification of HdcA, a histidine decarboxylase (HisDC). Additional factors like special chaperones might also be involved in pyruvoyl group formation in vivo [7].

### Conclusion

In this study, we developed a new method for predicting the protein post-translational pyruvoyl serine sites and analyzed the optimal features. By means of the feature selection algorithm, an optimal set of 9 features were selected by applying a window length of 17; these features were regarded as the ones that contributed significantly to the prediction of the post-translational self-processing modification. With the 9 optimal features selected, our approach achieved an overall *accuracy* of 93.75% and *MCC* of 0.8454. Analysis of the optimal feature set showed that the PSSM conversation scores contributed the most. Results from this study also supported the important effects of β-strand structures on pyruvoyl residue modifications. Since the mechanism for cysteine pyruvoyl modifications was similar to that of serine [7], these selected features may shed some light into in-depth understanding of the mechanism of not only pyruvoyl serine modifications but also other residue pyruvoyl modifications, providing guidelines for experimental validation. At last, the predicting software is available and can be downloaded from http://www.nkbiox.com/sub/pyrupred/index.html.

## Supporting Information

File S1 **The 4 datasets used in this study.** The accession numbers of the proteins and the positions of the center serine sites were given.
(XLS)

File S2 **Amino acid frequencies around the pyruvoyl serine sites in 21-residue peptides, which was the maximum window length used in this study.**
(XLS)

File S3 **The mRMR results of the 4 datasets when the window size was set to 17, with one sheet for one dataset.** Each sheet contains two tables. The first one is the MaxRel feature table ranking according to the relevance between the features and the class of the samples. The second one is the mRMR feature table ranking according to the relevance to the target and the redundancy among the features.
(XLS)

File S4 **The sensitivity (** ***Sn*** **), specificity (** ***Sp*** **), accuracy (** ***Ac*** **), Matthews correlation coefficient (** ***MCC*** **) generated by each run of the IFS when window length was set to 17 in the 4 datasets.**
(XLS)

## References

[1] Schmitzberger F, Kilkenny ML, Lobley CMC, Webb ME, Vinkovic M, et al (2003) Structural constraints on protein self-processing in L-aspartate-a-decarboxylase. The EMBO Journal 22(23): 6193–6204. 14633979 10.1093/emboj/cdg575 PMC291833

[2] Toms AV, Kinsland C, McCloskey DE, Pegg AE, Ealick SE (2004) Evolutionary Links as Revealed by the Structure of Thermotoga maritima S-Adenosylmethionine Decarboxylase. The Journal of Biological Chemistry 279(32): 33837–33846. 15150268 10.1074/jbc.M403369200

[3] Gerner EW, Meyskens FL Jr, Goldschmid S, Lance P, Pelot D (2007) Rationale for, and design of, a clinical trial targeting polyamine metabolism for colon cancer chemoprevention. Amino Acids 33: 189–195. 17396214 10.1007/s00726-007-0515-2

[4] Gerner EW, Meyskens FL Jr (2004) Polyamines and cancer: old molecules, new understanding. Nat Rev Cancer 4: 781–792. 15510159 10.1038/nrc1454

[5] Casero RA Jr, Marton LJ (2007) Targeting polyamine metabolism and function in cancer and other hyperproliferative diseases. Nat Rev Drug Discov 6: 373–390. 17464296 10.1038/nrd2243

[6] Bale S, Ealick SE (2010) Structural biology of S-adenosylmethionine decarboxylase. Amino Acids 38: 451–460. 19997761 10.1007/s00726-009-0404-y PMC2847442

[7] Bednarski B, Andreesen JR, Pich A (2001) In vitro processing of the proproteins grdE of protein B of glycine reductase and prdA of D-proline reductase from Clostridium sticklandii: formation of a pyruvoyl group from a cysteine residue. Eur.J.Biochem 268: 3538–3544. 11422384 10.1046/j.1432-1327.2001.02257.x

[8] Albert A, Dhanaraj V, Genschel U, Khan G, Ramjee MK, et al (1998) Crystal structure of aspartate decarboxylase at 2.2 A resolution provides evidence for an ester in protein self-processing. Nat Struct Biol 5: 289–293. 9546220 10.1038/nsb0498-289

[9] Webb ME, Lobley CMC, Soliman F, Kilkenny ML, Smith AG, et al (2012) Structure of Escherichia coli aspartate a-decarboxylase Asn72Ala: probing the role of Asn72 in pyruvoyl cofactor formation. Acta Crystallographica F68: 414–417. 10.1107/S1744309112009487 PMC3325809 22505409

[10] Hugo ER, Byers TJ (1993) S-Adenosyl-L-methionine decarboxylase of Acanthamoeba castellanii (Neff): purification and properties. Biochem.J 295: 203–209. 8216217 10.1042/bj2950203 PMC1134839

[11] Wada M, Shirahata A (2010) Identification of the Primary Structure and Post-translational Modification of Rat S-Adenosylmethionine Decarboxylase. Biol.Pharm. Bull 33(5): 891–894. 10.1248/bpb.33.891 20460772

[12] Ekstrom JL, Tolbert WD, Xiong H, Pegg AE, Ealick SE (2001) Structure of a human S-adenosylmethionine decarboxylase self-processing ester intermediate and mechanism of putrescine stimulation of processing as revealed by the H243A mutant. Biochemistry 40: 9495–9504. 11583148 10.1021/bi010736o

[13] Giles TN, Graham1 DE (2008) Crenarchaeal Arginine Decarboxylase Evolved from an S-Adenosylmethionine Decarboxylase Enzyme. The Journal of Biological Chemistry 283(38): 25829–25838. 18650422 10.1074/jbc.M802674200 PMC2533785

[14] Graham DE, Xu H, White RH (2002) Methanococcus jannaschii Uses a Pyruvoyl-dependent Arginine Decarboxylase in Polyamine Biosynthesis. The Journal of Biological Chemistry 277(26): 23500–23507. 11980912 10.1074/jbc.M203467200

[15] Pegg AE, Xiong H, Feith D, Shantz LM (1998) S-adenosylmethionine decarboxylase: structure, function and regulation by polyamines. Biochem Soc Trans 26: 580–586. 10047786 10.1042/bst0260580

[16] Smith CB, Graham DE (2008) Outer and Inner Membrane Proteins Compose an Arginine-Agmatine Exchange System in Chlamydophila pneumoniae. Journal of Bacteriology 190(22): 7431–7440. 18790867 10.1128/JB.00652-08 PMC2576674

[17] Lonvaud-Funel A (2001) Biogenic amines in wines: role of lactic acid bacteria. FEMS Microbiol Lett 199: 9–13. 11356560 10.1111/j.1574-6968.2001.tb10643.x

[18] Trip H, Mulder NL, Rattray FP, Lolkema JS (2011) HdcB a novel enzyme catalysing maturation of pyruvoyl-dependent histidine decarboxylase. Molecular Microbiology 79(4): 861–871. 21208300 10.1111/j.1365-2958.2010.07492.x

[19] Gallagher T, Snell EE, Hackert ML (1989) Pyruvoyl-dependent histidine decarboxylase. Active site structure and mechanistic analysis. J Biol Chem 264: 12737–12743. 2745463

[20] Li QX, Dowhan W (1990) Studies on the Mechanism of Formation of the Pyruvate Prosthetic Group of Phosphatidylserine Decarboxylase from Escherichia coli. The Journal of Biological Chemistry 265(7): 4111–4115. 2406271

[21] Basu S, Plewczynski D (2010) AMS3.0: prediction of post-translational modifications. BMC Bioinformatics 11: 210–224. 20423529 10.1186/1471-2105-11-210 PMC2874555

[22] Xue Y, Li A, Wang L, Feng H, Yao X (2006) PPSP: prediction of PK-specific phosphorylation site with Bayesian decision theory. BMC bioinformatics 7: 163. 16549034 10.1186/1471-2105-7-163 PMC1435943

[23] Blom N, Sicheritz-Ponten T, Gupta R, Gammeltoft S (2004) Brunak S: Prediction of post-translational glycosylation and phosphorylation of proteins from the amino acid sequence. Proteomics 4(6): 1633–1649. 15174133 10.1002/pmic.200300771

[24] Wong YH, Lee TY, Liang HK, Huang CM, Wang TY, et al.. (2007) KinasePhos 2.0: a web server for identifying protein kinase-specific phosphorylation sites based on sequences and coupling patterns. Nucleic acids research W588–594. 10.1093/nar/gkm322 PMC1933228 17517770

[25] Zhang N, Li BQ, Gao S, Ruan JS, Cai YD (2012) Computational prediction and analysis of protein γ-carboxylation sites based on a random forest method. Mol.BioSyst 8: 2946–2955. 22918520 10.1039/c2mb25185j

[26] Shao J, Xu D, Tsai SN, Wang Y (2009) Ngai SM: Computational identification of protein methylation sites through bi-profile Bayes feature extraction. PLoS One 4(3): e4920. 19290060 10.1371/journal.pone.0004920 PMC2654709

[27] Li S, Li H, Li M, Shyr Y, Xie L, et al (2009) Improved prediction of lysine acetylation by support vector machines. Protein Pept Lett 16(8): 977–983. 19689425 10.2174/092986609788923338

[28] Julenius K, Molgaard A, Gupta R, Brunak S (2005) Prediction, conservation analysis, and structural characterization of mammalian mucin-type Oglycosylation sitess. Glycobiology 15(2): 153–164. 15385431 10.1093/glycob/cwh151

[29] Hamby SE, Hirst JD (2008) Prediction of glycosylation sites using random forests. BMC bioinformatics 9: 500. 19038042 10.1186/1471-2105-9-500 PMC2651179

[30] Li BQ, Hu LL, Niu S, Cai YD, Chou KC (2012) Predict and analyze S-nitrosylation modification sites with the mRMR and IFS approaches. Journal of Proteomics 75(5): 1654–1665. 22178444 10.1016/j.jprot.2011.12.003

[31] Apweiler R, Martin MJ, O'Donovan C, Magrane M, Alam-Faruque Y, et al (2010) The Universal Protein Resource (UniProt) in 2010. Nucleic Acids Res 38: D142–8. 19843607 10.1093/nar/gkp846 PMC2808944

[32] Altschul SF, Madden TL, Schaffer AA, Zhang J, Zhang Z, et al (1997) Gapped BLAST and PSI-BLAST: a new generation of protein database search programs. Nucleic Acids Res 25: 3389–402. 9254694 10.1093/nar/25.17.3389 PMC146917

[33] Ferron F, Longhi S, Canard B, Karlin D (2006) A Practical Overview of Protein Disorder Prediction Methods. PROTEINS:Structure, Function, and Bioinformatics 65: 1–14. 10.1002/prot.21075 16856179

[34] Noivirt-Brik O, Prilusky J, Sussman JL (2009) Assessment of disorder predictions in CASP8. Proteins 77(Suppl9): 210–216. 19774619 10.1002/prot.22586

[35] Peng K, Radivojac P, Vucetic S, Dunker AK, Obradovic Z (2006) Length-dependent prediction of protein intrinsic disorder. BMC Bioinformatics 7: 208. 16618368 10.1186/1471-2105-7-208 PMC1479845

[36] Cheng J, Randall AZ, Sweredoski MJ, Baldi P (2005) SCRATCH: a protein structure and structural feature prediction server. Nucleic Acids Res 33: W72–6. 15980571 10.1093/nar/gki396 PMC1160157

[37] Kawashima S, Kanehisa M (2000) AAindex: amino acid index database. Nucleic Acids Res 28: 374. 10592278 10.1093/nar/28.1.374 PMC102411

[38] Atchley WR, Zhao J, Fernandes AD, Druke T (2005) Solving the protein sequence metric problem. Proc Natl Acad Sci U S A 102: 6395–400. 15851683 10.1073/pnas.0408677102 PMC1088356

[39] Peng H, Long F, Ding C (2005) Feature selection based on mutual information: criteria of max-dependency. max-relevance,and min-redundancy.IEEE Trans Pattern Anal Mach Intell 27: 1226–38. 16119262 10.1109/TPAMI.2005.159

[40] Li BQ, Hu LL, Chen L, Feng KY, Cai YD, et al (2012) Prediction of protein domain with mRMR feature selection and analysis. PLoS One 7(6): e39308. 22720092 10.1371/journal.pone.0039308 PMC3376124

[41] Li BQ, Huang T, Liu L, Cai YD, Chou KC (2012) Identification of colorectal cancer related genes with mRMR and shortest path in protein-protein interaction network. PLoS One 7(4): e33393. 22496748 10.1371/journal.pone.0033393 PMC3319543

[42] He Z, Zhang J, Shi XH, Hu LL, Kong X, et al (2010) Predicting drug-target interaction networks based on functional groups and biological features. PLoS One 5 e9603. 20300175 10.1371/journal.pone.0009603 PMC2836373

[43] Huang T, Cui W, Hu L, Feng K, Li YX, et al (2009) Prediction of pharmacological and xenobiotic responses to drugs based on time course gene expression profiles. PLoS One 4: e8126. 19956587 10.1371/journal.pone.0008126 PMC2780314

[44] Breiman L (2001) Random forests. Mach learn 45: 5–32.

[45] Jia SC, Hu XZ (2011) Using random forest algorithm to predict beta-hairpin motifs. Protein Pept Lett 18: 609–17. 21309739 10.2174/092986611795222777

[46] Shameer K, Pugalenthi G, Kandaswamy KK, Sowdhamini R (2011) 3dswap-pred: prediction of 3D domain swapping from protein sequence using random forest approach. Protein Pept Lett 18: 1010–20. 21592079 10.2174/092986611796378729

[47] Rogers J, Gunn S (2006) Identifying feature relevance using a random forest. Subspace,Latent Struct Feature Sel 3940: 173–84.

[48] Witten IH, Frank E (2005) Data Mining: Practical Machine Learning Tools and Techniques. 2nd Edition. San Francisco: Morgan Kaufmann.

[49] He B, Wang K, Liu Y, Xue B, Uversky VN, et al (2009) Predicting intrinsic disorder in proteins: an overview. Cell Res 19: 929–949. 19597536 10.1038/cr.2009.87

[50] Li BQ, Cai YD, Feng KY, Zhao GJ (2012) Prediction of Protein Cleavage Site with Feature Selection by Random Forest. PLoS ONE 7(9): e45854. 23029276 10.1371/journal.pone.0045854 PMC3445488

[51] Tyndall JD, Nall T, Fairlie DP (2005) Proteases universally recognize beta strands in their active sites. Chem Rev 105: 973–999. 15755082 10.1021/cr040669e

[52] Fairlie DP, Tyndall JD, Reid RC, Wong AK, Abbenante G, et al (2000) Conformational selection of inhibitors and substrates by proteolytic enzymes: implications for drug design and polypeptide processing. J Med Chem 43: 1271–1281. 10753465 10.1021/jm990315t

[53] You L (2006) Detection of cleavage sites for HIV-1 protease in native proteins. Comput Syst Bioinformatics Conf: 249–256. 17369643

[54] Fooks HM, Martin AC, Woolfson DN, Woolfson DN, Sessions RB, et al (2006) Amino Acid Pairing Preferences in Parallel b-Sheets in Proteins. J.Mol.Biol 356: 32–44. 16337654 10.1016/j.jmb.2005.11.008

[55] Zhang N, Ruan JS, Duan GY, Gao S, Zhang T (2009) The interstrand amino acid pairs play a significant role in determining the parallel or antiparallel orientation of b-strands. Biochemical and Biophysical Research Communications 386: 537–543. 19540200 10.1016/j.bbrc.2009.06.072

[56] Zhang N, Duan GY, Gao S, Ruan JS, Zhang T (2010) Prediction of the parallel/antiparallel orientation of beta-strands using amino acid pairing preferences and support vector machines. Journal of Theoretical Biology 263(3): 360–368. 20035768 10.1016/j.jtbi.2009.12.019

